# Unveiling the structure, function and dynamics of StmPr1 in *Stenotrophomonas maltophilia* virulence

**DOI:** 10.1038/s41598-025-06177-5

**Published:** 2025-06-20

**Authors:** Max Sommer, Amr Negm, Lasse Outzen, Sabine Windhorst, Azat Gabdulkhakov, Wolfgang Weber, Christian Betzel

**Affiliations:** 1 Department of Chemistry, Institute of Biochemistry and Molecular Biology, Laboratory for Structural Biology of Infection and Inflammation, University of Hamburg, c/o DESY, Build. 22a. Notkestr. 85, 22603 Hamburg, Germany; 2 Department of Chemistry, College of Science, King Faisal University, 31982 Al-Ahsa, Saudi Arabia; 3 Chemistry Department, Faculty of Science, Mansoura University, Mansoura, 35516 Egypt; 4 Department of Chemistry, Institute of Pharmacy, University of Hamburg, Bundesstraße 45, 20146 Hamburg, Germany; 5 Institute for Biochemistry and Signal transduction, University Medical Center Hamburg-Eppendorf, University Medical Center Hamburg Eppendorf, Martinistraße 52, 20246 Hamburg, Germany; 6 Institute of Protein Research, Russian Academy of Sciences, Pushchino, Russia; 7 Institute of Biochemistry and Molecular Biology I, University Medical Center Hamburg- Eppendorf, Martinistr. 52, 20246 Hamburg, Germany

**Keywords:** *Stenotrophomonas maltophilia*, Serine protease StmPr1, Structural biology, Nosocomial infections, Drug discovery, Bortezomib Inhibition, Structural biology, Diseases

## Abstract

The increase in infections caused by multi-resistant Gram-negative bacteria, like *Stenotrophomonas maltophilia*, has become a growing health crisis worldwide. *S. maltophilia* poses a risk because of its tendency to opportunistically infecting patience for example through colonization of catheters in hospital environments using its intrinsic resistance against multiple antibiotics. Through the COVID-19 pandemic it gained more prominence by being a key pathogen in respiratory co-infections. This study will present a structural analysis of StmPr1, *S. maltophilia*’s main virulence factor, an excreted serine protease. Our study outline structure and functional aspects of StmPr1, revealing a unique autoproteolytic activity resulting in a shortened version of the active enzyme. We also investigated the potential of two groups of peptide-based inhibitors, one being acetyl- and the other being boron-based inhibitors. The focus here lies on Bortezomib, a boron-based serine protease inhibitor, and its potential therapeutic use against *S. maltophilia.* We provide a structure-function analysis which includes X-ray crystallography data with resolutions ranging from 1.64 to 2.08 Å, molecular dynamic simulations and small-angle X-ray scattering (SAXS) experiments. These data provide a deeper understanding of StmPr1’s resilience and mechanisms, while highlighting the relevance of StmPr1’s C-terminal extension for correct folding and its stability. Moreover, it also shows that StmPr1 is promising target for further drug discovery investigations to identify compounds and drugs to treat *S. maltophilia* infections.

**Supplementary Information:**

The online version contains supplementary material available at 10.1038/s41598-025-06177-5.

## Introduction

The globally rising number of deaths related to multi-resistant Gram-negative bacteria has emerged to be one of the most challenging clinical and public health problems during the last years^1,2^. While immense progress in pharmaceutical and drug discovery research has been achieved, it remains a challenge to treat several distinct infectious diseases. Particularly nosocomial infections caused by opportunistic Gram-negative pathogens are increasingly difficult to treat, caused by rising intrinsic and acquired antibiotic resistance^3^. A Gram-negative multi-resistant opportunistic pathogen of increasing relevance is *Stenotrophomonas maltophilia. S. maltophilia* is a non-fermenting Gram-negative bacillus that can be isolated globally. It is found in different environmental sources, such as water, soil, sediment, plants, and animals^4–8^. Despite not being inherently virulent, it causes serious infections in immunocompromised patients, including pneumonia, catheter-associated bacteremia/septicemia, osteochondritis, mastoiditis, meningitis, and endocarditis. It is associated with crude mortality rates ranging from approx. 15% to 70% in bacteremia patients^9–12^. Due to its role in nosocomial infections, *S. maltophilia* has increasingly become the focus of biomedical research over the last two decades. Reported *S. maltophilia* cases are rising considerably, becoming the most common Gram-negative carbapenem-resistant pathogen found in bacteremia particularly in hospitals^13,14^. Besides carbapenem, it is intrinsically resistant to multiple antibiotics, including multiple broad-spectrum antibiotics^12^. The bacterium is frequently found in patients with cystic fibrosis, with the frequency ranging from 10 to 30%^15^. During the COVID-19 pandemic, *S. maltophilia* was identified to be the main pathogen involved in respiratory co-infections and bacteremia in critically ill COVID-19 patients. *S. maltophilia* found in sputum samples of such patients showed the highest rates of multidrug resistance among bacteria infecting COVID-19 patients^16–18^. This role in polymicrobial bacterial communities is of increasing relevance given its ability to influence neighboring microorganisms’ metabolism by antagonistic suppression or by symbiotic coexistence. *S. maltophilia* colonizes cystic fibrosis patients alongside *Pseudomonas aeruginosa*, *Staphylococcus aureus*, or *Burkholderia cenocepacia*^19,20^. Besides its role in polymicrobial bacterial communities, *S. maltophilia* shows a broad spectrum of virulence factors consisting of surface cell-associated structures and a wide spectrum of extracellular enzymes^21^. Our investigation focused on obtaining insights into the structure-function-relationship of one of the main extracellular enzymes, the secretory protease StmPr1.

StmPr1 is a subtilisin-like serine protease^22^. In general, those subtilases are globular proteins with a molecular weight of approx. 27 kDa. Interestingly, the gene of the main protease *stmpr1* codes for a 63 kDa precursor, which is processed to a mature protein of 47 kDa. The main reason for the size difference is a C-terminal extension normally not found in other subtilases. We also found that the 47 kDA protein digests itself to a 36 kDa protein over a period of approx. 7 days. Furthermore, we investigate the potential of Bortezomib, a boron-based pseudopeptide serine protease inhibitor already used in cancer treatment, for its potential use as an alternative in *S. maltophilia* treatment^23^.

## Results and discussion

### StmPr1 36 kda structure analysis

**Fig. 1:**
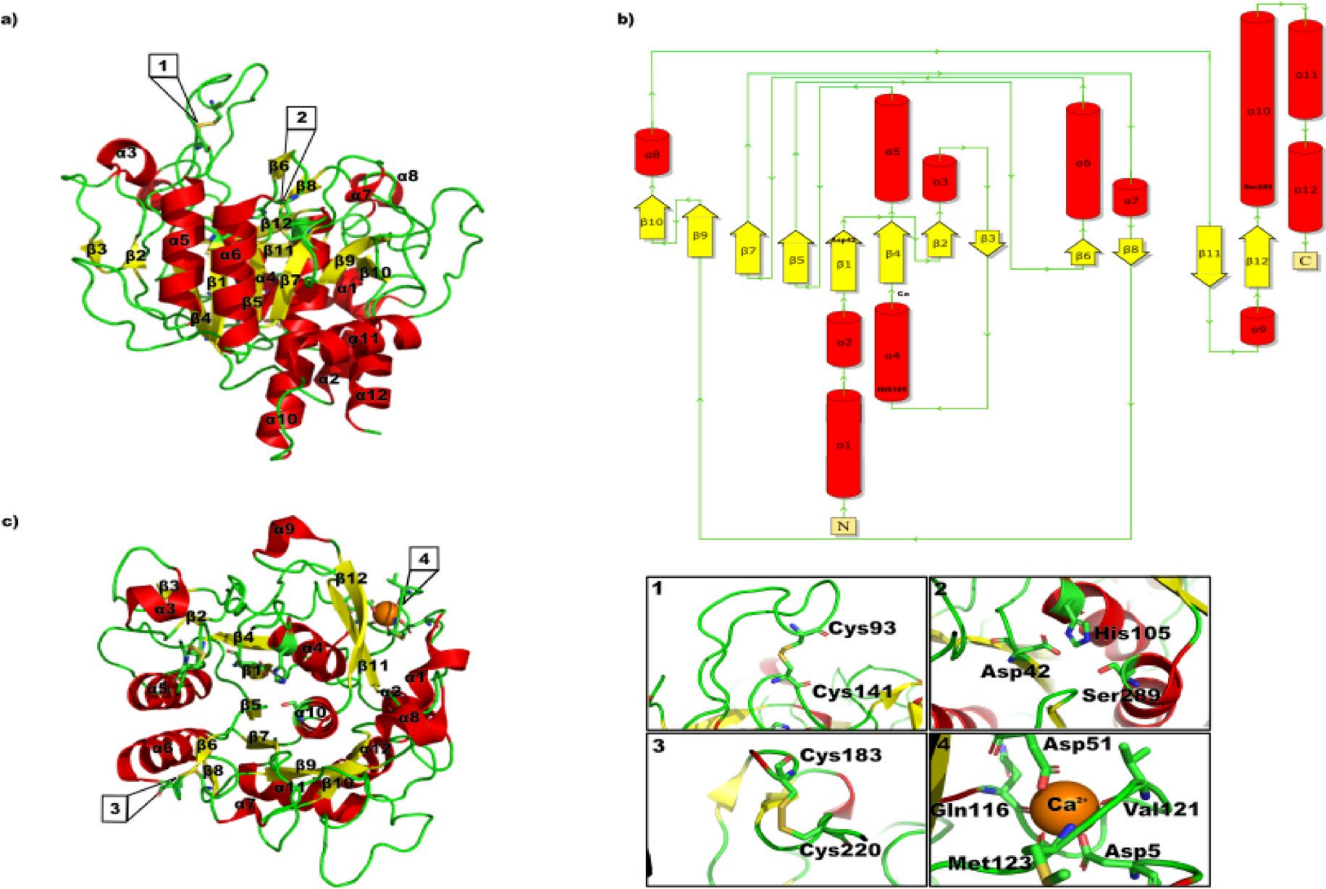
Cartoon and topology plot of StmPr1 36 kDa. (a) The secondary structure, solved by X-ray crystallography, is indicated in different colors. Loops are depicted in green, sheets in yellow and helices in red. (b) The topology plot is depicted in the same color scheme and highlights the Ca-binding site and catalytic triad. (c) Top view of the secondary structure indicated in the same colors to show secondary structure features missing in a). The structure is rotated by 90° with the active site on top. Inserts 1–4 highlight relevant structural elements of StmPr1. The two disulfide bridges (Cys93-Cys141, 1 and Cys183 - Cys220, 3), the catalytic triad (2) and the calcium binding site (4) are highlighted in their own window.

**Fig. 2:**
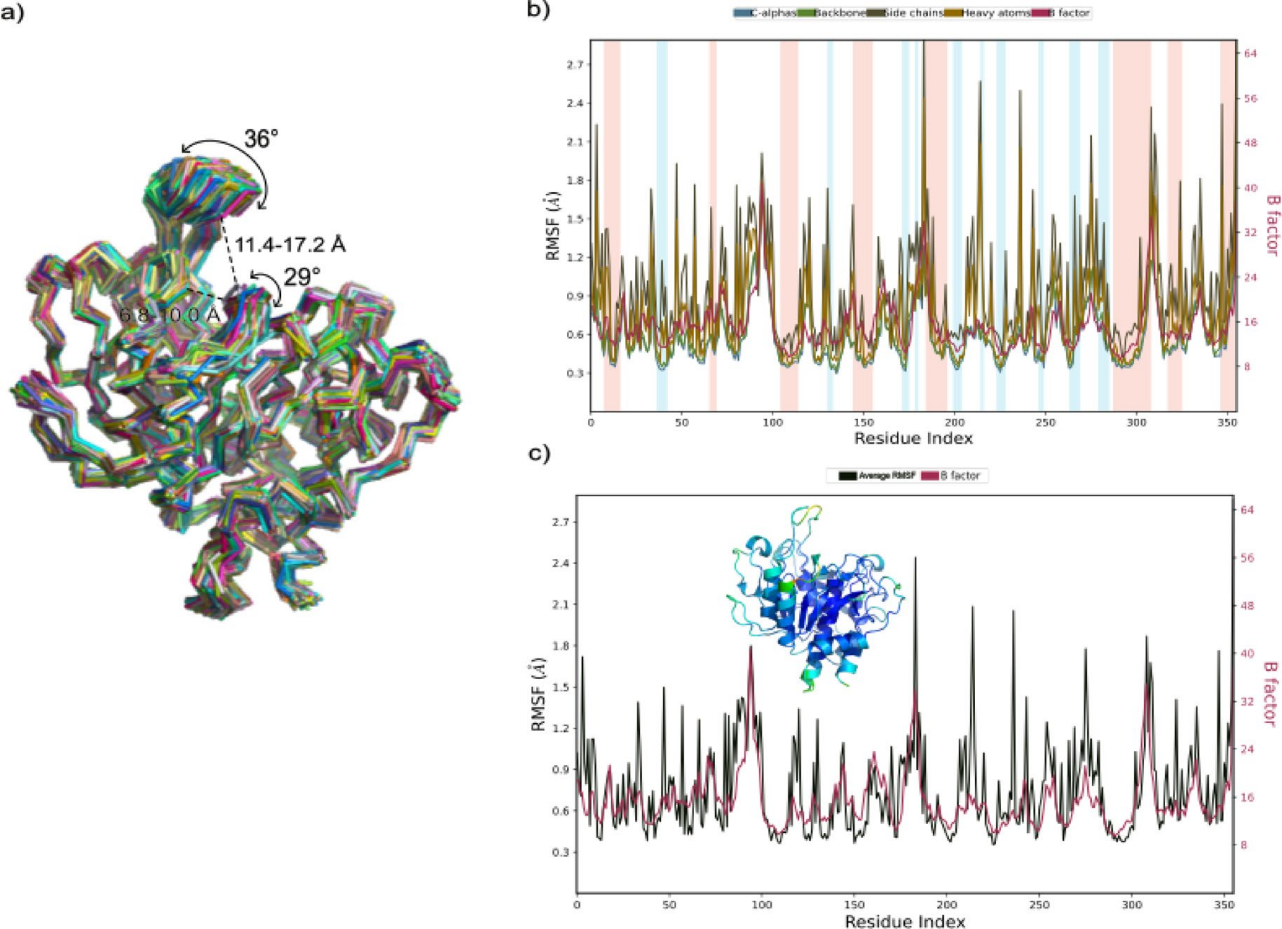
Flexibility analysis of StmPr1 36 kDa, Molecular Dynamics Simulation ran for 100 ns. (a) 250 frames extracted over the simulation superimposed. Delineations of flexible and rigid regions of the lid region over the catalytic triad are annotated. (b) Root Mean Square Fluctuation (RMSF) and B-factor plots displaying changes in Cα, backbone, side chains, and heavy atoms for each residue. Different colors show different secondary structure elements: α-helices, blue, and β-sheets, pink. (c) Average B-factor of diffraction data compared with RMSF values of the MD simulation. The cartoon representation of StmPr1 36 kDa is color-coded to emphasize variations in B-factor values, with colder colors representing lower B-factor values, further highlighting structural dynamic. Simulations were done with Schoedinger’s Maestro Suite^33^.

**Fig. 3:**
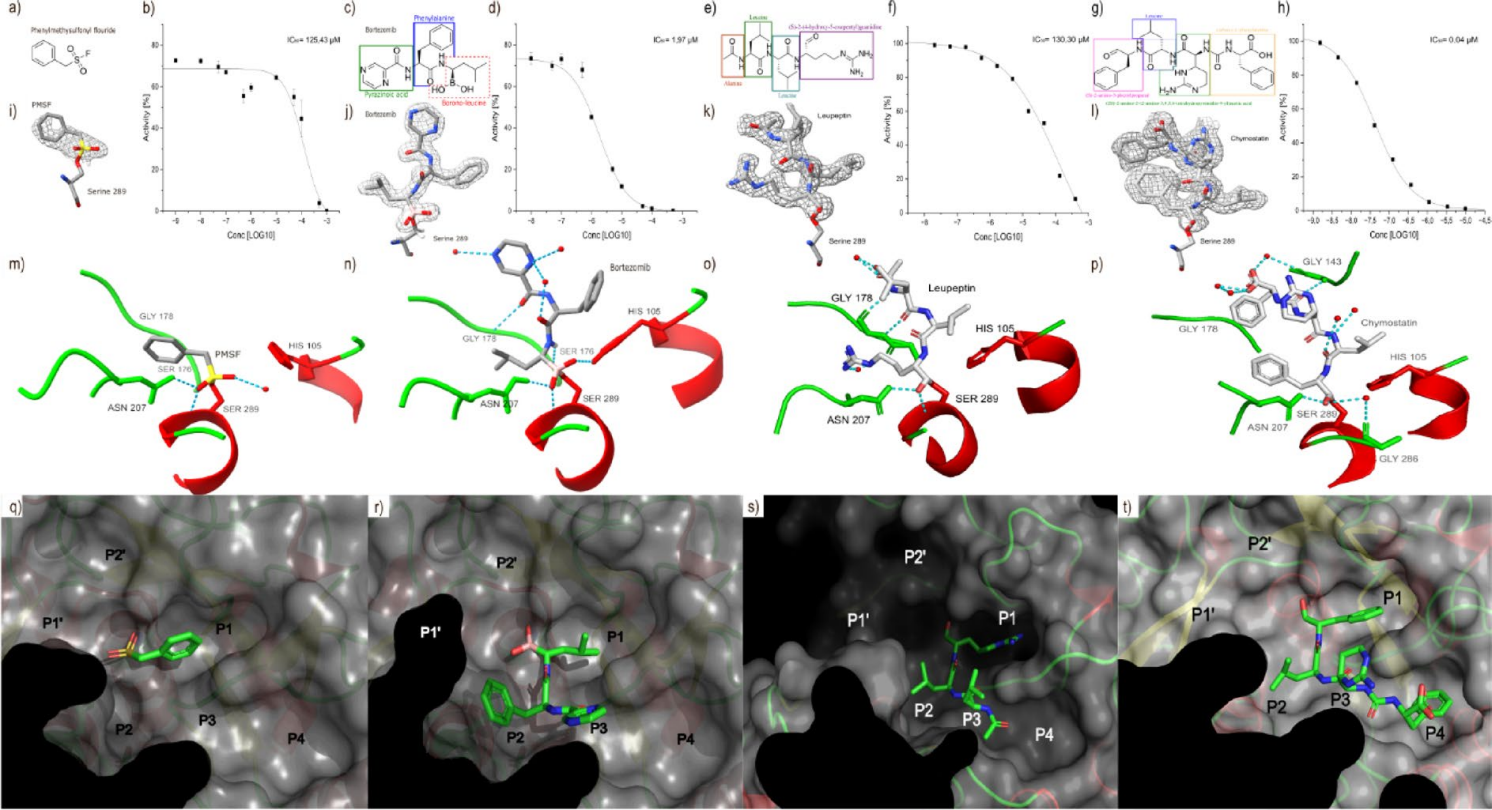
Binding characterization of PMSF, Bortezomib, Leupeptin and Chymostatin with StmPr1 36 kDa. Panel a) shows the 2D structure of Phenylmethyl sulfonyl fluoride (PMSF), c) shows the 2D structure of Bortezomib, e) shows the 2D structure of Leupeptin and g) shows the 2D structure of Chymostatin. Under that the ligands PMSF (i), Bortezomib (j), Leupeptin(k) and Chymostatin (l) are embedded into the electron density at sigma level 2.0 in the omit map. Beside that activity curves show StmPr1 36 kDa’s activity and IC_50_ against PMSF (b), Bortezomib (d), Leupeptin (f) and Chymostatin (h). Dashed lines represent the hydrogen bonds for (m) PMSF, (n) Bortezomib, (o) Leupeptin and (p) Chymostatin. Below that are the substrate binding cleft with PMSF (q), Bortezomib (r), Leupeptin (s) and Chymostatin (t).

StmPr1’s 36 kDa structure, solved and analyzed by x-ray crystallography, shares structural similarities with other subtilases, first described by Wright et al. for subtilisin BPN’^24^. The catalytic triad consisting of Asp42, His105, and Ser289 is located on top of β1 (Asp42), α4 (His105), and α10 (Ser289) (Figs. 1a and 2). Ser289 also forms the oxyanion hole together with Asp207 stabilizing the tetrahedral transition state shown for PMSF and Bortezomib in Fig. 3g and h. StmPr1 has the subtilase-typical α/β fold formed by seven, instead of eight, parallel β-sheets with five α-helices surrounding the sheets where four helices are antiparallel to the sheets. While α4, α5, α6, and α10 surround the sheets in an antiparallel manner, α11 supports the structure stability from the bottom (Fig. 1a & c). Of the eight sheets, number 3 is antiparallel, whereas β1, β2, β4, β5, β7, β9, and β10 form the parallel core (Fig. 1b). Whilst the parallel structures increase to some extent flexibility and allow better adaption to several substrates, the antiparallel conformation on the periphery of the protein increases its stability^25^. Besides the seven parallel β-sheets forming the core, StmPr1 shows only antiparallel β-sheet formations in β2/β3, β6/β8, and β11/β12, all located on the fringes of the structure and therefore supporting the structural stability in most loop-rich regions of the protein. The β11/β12 formation is a structure also found in other subtilases like subtilisin BPN’^24^ Proteinase K^26^ or Thermitase^27^ supporting the 3D structure of the substrate binding cleft (Figs. 1c and 3i and j).

However, none of the aforementioned shares β6/β8 with StmPr1. Despite being only short sheets, two amino acids each, β6/β8 supports key regions with β6 located between the connection of β5 and α6 and β8 being located in between β7 and β9 (Fig. 1). This loop is also shorter compared to subtilisin BPN’24 and subtilisin Carlsberg^28^ matching the structure of Proteinase K^26^ and Thermitase^27^. A further structure separating the element of StmPr1 from the other subtilases is α7, a short helix close to β8. StmPr1 shares both features with the cold-active serine protease Pro21717^29^. Both adaptations most likely expand the temperature tolerance by increasing the stability of the surface loop^30,31^.

Another adaptation affecting thermostability is the region around Asp5, Asp51, Gln116, Asn119, Val121 and Met123. These residues form a calcium-binding site, coordinating a single Ca²⁺ ion in a pentagonal bipyramidal arrangement through the carboxyl groups of Asp5 and Asp51, as well as the oxygen atoms of Gln116, Val121, and Met123 and the carbamoyl group of Asn119, which also contributes to local stability. Furthermore, Richardson et al. postulates that intracellular calcium levels play a role in preventing intracellular proteolysis by being too low to produce an active protein^32^. This represents a mechanism relevant to a broad-spectrum serine protease like StmPr1^22^.

The protein also has two disulfide bonds, Cys93 - Cys141 and Cys183 - Cys220. The latter of which connects the earlier described loop connecting β5 and α6 to the loop connecting β7 and β9, additionally strengthening this region. Cys93-Cys141 links the loop connecting β1 and α5 to a loop covering the catalytic triad, beginning directly behind β3 at Gly79 and going to Ser103 on top of α4. Thereby, it strengthens the connection of the loop to the core structure helping to form a kind of lid above the substrate binding cleft. This lid is a loop insertion, not present in subtilisin BPN’^24^ Proteinase K^26^ Thermitase^27^ or subtilisin Carlsberg^28^. Nevertheless, StmPr1 shares this loop insertion with Pro21717^29^. The lid may facilitate and support two distinct functions, on the one hand, the lid may move to block the entry into the substrate binding cleft, thereby preventing unwanted binding to the catalytic triad. Thus, dampening the effectiveness of possible inhibitors. On the other hand, closing it also stabilizes potential substrates inside the substrate-binding cleft. This increases proteolytic activity, and also potentially widens substrate specificity by retaining less-suited substrates. Further insight into this mechanism comes from understanding the lid’s movement in solution. To gain this insight, we performed molecular dynamics simulations of StmPr1 using the crystallographically obtained structure as a model. The StmPr1 protease exhibits some special and additional structural features, like a relatively high content of Glu and Asp residues and a relatively low content of Arg and Lys residues on the surface, which may give the enzyme the ability to be active in an alkaline environment. This corresponds to the observation that StmPr1 is classified as an alkaline protease with an optimum pH of 9.

**Table 1:** X-ray data collections and refinements.

	StmPr1	StmPr1-PMSF	StmPr1-Bortezomib	StmPr1 Leupeptin	StmPr1 Chymostatin
Data collection					
X-ray source	P13, Petra III, DESY	P11, Petra III, DESY	P11, Petra III, DESY	X13, DORIS, Consortium-Beam Line, DESY	X13, DORIS, Consortium-Beam Line, DESY
Detector	EIGER 16 M	PILATUS 6 M-F	PILATUS 6 M-F	Mar CCD 165 mm	Mar CCD 165 mm
Space group	C2 2 2_1_	C2 2 2_1_	C2 2 2_1_	C2 2 2_1_	C2 2 2_1_
Cell dimensions					
a, b, c (Å)	60.38 86.38 131.57	59.90 85.75 130.73	60.44 86.75 131.30	60.78 86.41 132.35	60.43 86.51 132.20
Wavelength (Å)	0.96800	1.0332	1.0332	0.8123	0.8123
Resolution range (Å)	49.49–1.64 (1.70–1.64)	49.15–1.89 (1.96–1.89)	46.4–1.89 (1.96–1.89)	30.0–2.08 (2.15–2.08)	30.0–1.98 (2.06–1.99)
Total Nr. of reflections	575,348 (27769)	391,319 (37395)	395,217 (38294)	102,137 (12424)	144,330 (17280)
Total unique reflections	42,779 (2107)	54,073 (5384)	55,179 (5520)	21,042 (2742)	24,332 (3272)
Wilson B-factor (Å^2^)	17.50	19.32	25.88	14.86	19.40
R_meas_	0.185 (1.376)	0.121 (0.446)	0.151 (0.636)	0.164 (0.569)	0.129 (0.457)
I/σI	2.27	11.6	10.8	8.9	11.3
Completeness	100 (100)	99.7 (99.7)	99.0 (99.9)	98.2 (88.5)	98.8 (92.2)
Refinement					
Reflections used	42,779 (2140)	27,330 (1062)	55,144 (1094)	20,997 (1028)	23,860 (1171)
R_Work_	0.173 (0.261)	0.175 (0.539)	0.206 (0.255)	0.168 (0.24)	0.151 (0.259)
R_Free_	0.209 (0.295)	0.235 (0.562)	0.242 (0.268)	0.218 (0.335)	0.213 (0.284)
No. atoms					
Protein	2557	2528	2533	2546	2734
Ligand/ion	28	55	73	61	101
Water	377	319	337	145	293
Average B-Factor (Å^2^)					
Protein	18.14	26.63	28.26	17.17	17.5
Ligand/ion	42	53.21	50.59	37.7	39.47
Water	30.9	37.62	39.89	24.37	29.29
R.m.s deviations					
Bond lengths (Å)	0.0125	0.0081	0.0073	0.0221	0.023
Bond angles (°)	1.85	1.719	1.513	0.940	1.73
Ramachandran					
Favored (%)	98.02	96.33	96.89	90.5	90.5
Allowed (%)	1.98	2.49	2.72	9.5	9.5
Outliers (%)	0	1.18	0.39	0	0
PDB codes	9G8V	9GOI	9GRG	9I6C	9I67

### Molecular dynamics simulation analysis of StmPr1 36 kda

To further analyze the internal movement of Stmpr1 36 kDa a molecular dynamics simulation was performed, simulating how the structure behaves for 100 ns under physiological conditions (310.15 K, solvent water with 0.15 M sodium chloride) (Fig. 2). The RMSF values match the B-factor values of the crystallographic structure (Fig. 2b & c), showing a stable core with only small variations. Peripheral structures are more flexible in both models (Fig. 2c), especially the already discussed loop regions between β5 and α6, as well as the lid loop connecting β3 and α4. The β5/α6 loop moves through the simulation over a range of approx. 30°, while the β3/α4 lid covers a range of approx. 35°. Such movements change the distance between Tyr94/Ser180 by a total of 4.8 Å, effectively covering P2 and P3 of the substrate binding cleft. Whereas the amino acids Gly142 and Gly179 close the gap between each other for approx. 1/3 by 3.2 Å, thereby covering the P1 and P4 position.

This most likely would increase the binding of any substrate bond to the protein by covering the binding cleft but also may hinder potential inhibitors from binding to the catalytic triad or other positions inside the cleft^34^. Despite this being presumably a cyclic opening and closing, offering windows in which a substrate or inhibitor may bind, it shows a much stronger defense and trap mechanism compared to the other subtilisins not showing such adaptations. The fact that these adaptations appear only in other temperature-specialized proteases, such as Pro21717^29^, likely arises from their potential advantage in stabilizing proteolytic activity when temperature fluctuations alter molecular movement. At higher temperatures, they help to trap the substrate, despite increased molecular motion, while at lower temperatures, they can create a kind of flow that guides the substrate into the substrate-binding cleft despite slower molecular movement^35^.

### Inhibitors of StmPr1

To understand the interaction of StmPr1 with inhibitors, we investigated structures of StmPr1 in complex with the well-known protease inhibitor PMSF and the peptide aldehyde inhibitors Leupeptin and Chymostatin, as well as the peptide-like boron-based inhibitor Bortezomib (Fig. 3). Bortezomib is a proteasome inhibitor, using a boronic acid residue to reversibly bind at the active serine of serine proteases. It is used in the treatment of multiple myeloma and mantle cell lymphoma. There it is used in monotherapy or combination therapy^23^.

Accommodating the low solubility of ammonium sulfate in dimethyl sulfoxide, the solvent for Bortezomib, we adjusted the crystallization condition. Instead of 1.8 M ammonium sulfate, 0.1 M Tris, and a pH of 8, we used 0.9 M ammonium sulfate, 0.4 M lithium sulfate, and 0.1 M sodium citrate at pH 6. Thus, we lowered the ammonium sulfate concentration and pH to a point where occasional precipitation doesn’t affect the soaking and fishing of the crystals too much. Despite the changed conditions the resulting crystals show the same cell constants and diffracted to a similar resolution (Table 1). All inhibitors bind covalently to Ser289, forming a tetrahedral transition state supported by the main chain amid of Ser289 and the sidechain amino group of Asp207. PMSF is further stabilized by a water molecule and Bortezomib is stabilized by the catalytic triad His105 (Fig. 3m & n). Additionally, Bortezomib is stabilized at its position by Ser176 forming a H-bond to the nitrogen of the phenylalanine part of Bortezomib. The oxygen of the peptide bond between pyrazinoic acid and phenylalanine connects via H-bond to Gly178. Moreover, three water molecules stabilize Bortezomib through H-bonds with the nitrogen of the pyrazinoic acid and oxygen of the peptide bond connecting phenylalanine and borono-leucine (Fig. 3c & n). The complex with Leupeptin is supported by a water molecule mediated H-bonds at its guanidine residue and the oxygen of its alanine residue. Furthermore, it forms a hydrogen bond with Gly178 (Fig. 3f & o). The binding of Chymostatin is supported via H-bonds to Gly286 and Gly143. Water further stabilizes Chymostatin at its propanal and acetic acid residue (Fig. 3g & p).

Besides the covalent bond to Ser289, the phenyl part of PMSF is coordinated into the P1 site of the substrate binding cleft. Leupeptin shows a similar coordination with its guanidine residue located in P1, while the remaining part of the molecule extents over P2, with one leucine, to P3 being occupied by the other leucine and the alanine residue. For Bortezomib the P1 site is occupied by the methyl group of borono-leucine. The phenylalanine is located in the P2 site, while the pyrazinoic acid occupies P3.

Chymostatin shows a similar occupation pattern but extends further with its phenylalanine residue located in P4. This stronger bond also explains why Bortezomib and Chymostatin show much higher potential as inhibitors for StmPr1. While PMSF shows a IC_50_ of 125,43 µM needing a concentration of 1 mM to totally eradicate enzymatic activity and Leupeptin even shows a higher IC50 with 130,3 µM, Bortezomib has an IC_50_ of 1,97 µM, eradicating the activity at 100 µM and Chymostatin has an IC_50_ of 0,04 µM and needs 10 µM for total inhibition (Fig. 3b, d, f & h). The even lower IC_50_ of Chymostatin most likely stems from two main differences, while P1 is occupied with Bortezomib by a methyl group, Chymostatin binds to the P1 with a phenol ring further stabilizing the oxyanion hole. Furthermore, Chymostatin extends also into the P4 site, while Bortezomib only reaches the P3 site. While the lower IC_50_ highlights Chymostatins as a potential inhibitor for StmPr1, Bortezomib has the advantage of being an already approved medical drug, which is already in use as a proteasome inhibitor in cancer treatment. Therefore, Bortezomib is a solid inhibitor for StmPr1 using the combination of the pseudo-peptide structure with the strong binding capacity of boronic acid to block the catalytic triad by binding the active serine covalently, while the bond is supported by His105. Meanwhile, the peptide-like structure of Bortezomib blocks the P1, P2, and P3 sites of the substrate binding cleft. To compare StmPr1 with a subtilase, not showing the combinations structure of β5/α6 loop and β3/α4 lid, we did the same assays for Bortezomib with Proteinase K (Supplemental information). Proteinase K missing those adaptations has an IC_50_ of 1.45 µM and 40 µM Bortezomib is sufficient to stop the enzyme activity entirely. This further supports the proposed idea, that the adaptations of StmPr1 increase its resistance against inhibitors compared to other subtilases missing the lid structure formed by β5/α6 and β3/α4.

### StmPr1’s place in the subtilisin family

**Fig. 4:**
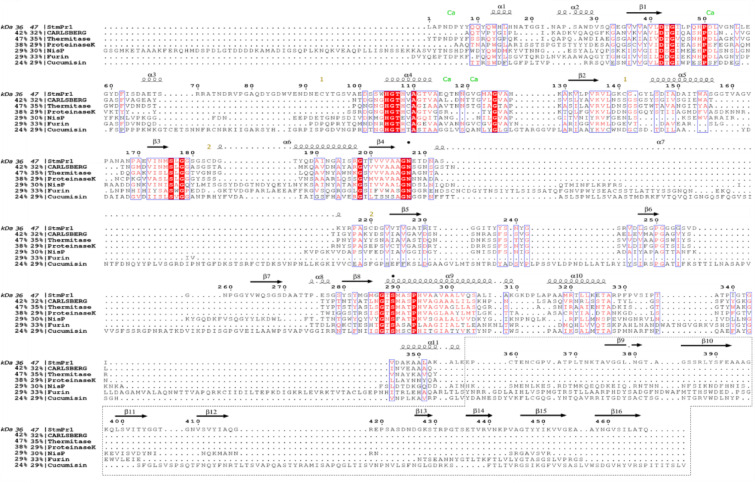
Sequence alignment of StmPr1 36 kDa and 47 kDa, Subtilisin Carlsberg from Bacillus licheniformis, Themitase from Thermoactinomyces vulgaris, Proteinase K from Tritirachium album limber, NisP from Lactococcus lactis, Furin from Mus Musculus and Cucumisin from Cucumis melo. Percentile values showing the structural identity of the proteins using the DALI server^37^. Identical amino acids are highlighted in red. Secondary structure elements of StmPr1 are indicated on top of the sequences. Calcium binding amino acids are marked with a green CA and the oxyanion hole is marked with black cycles. A dotted box highlights the PPC region unique to 47 kDa StmPr1. The figure was adopted using Figures created by ESPript 3.0^36^.

**Fig. 5:**
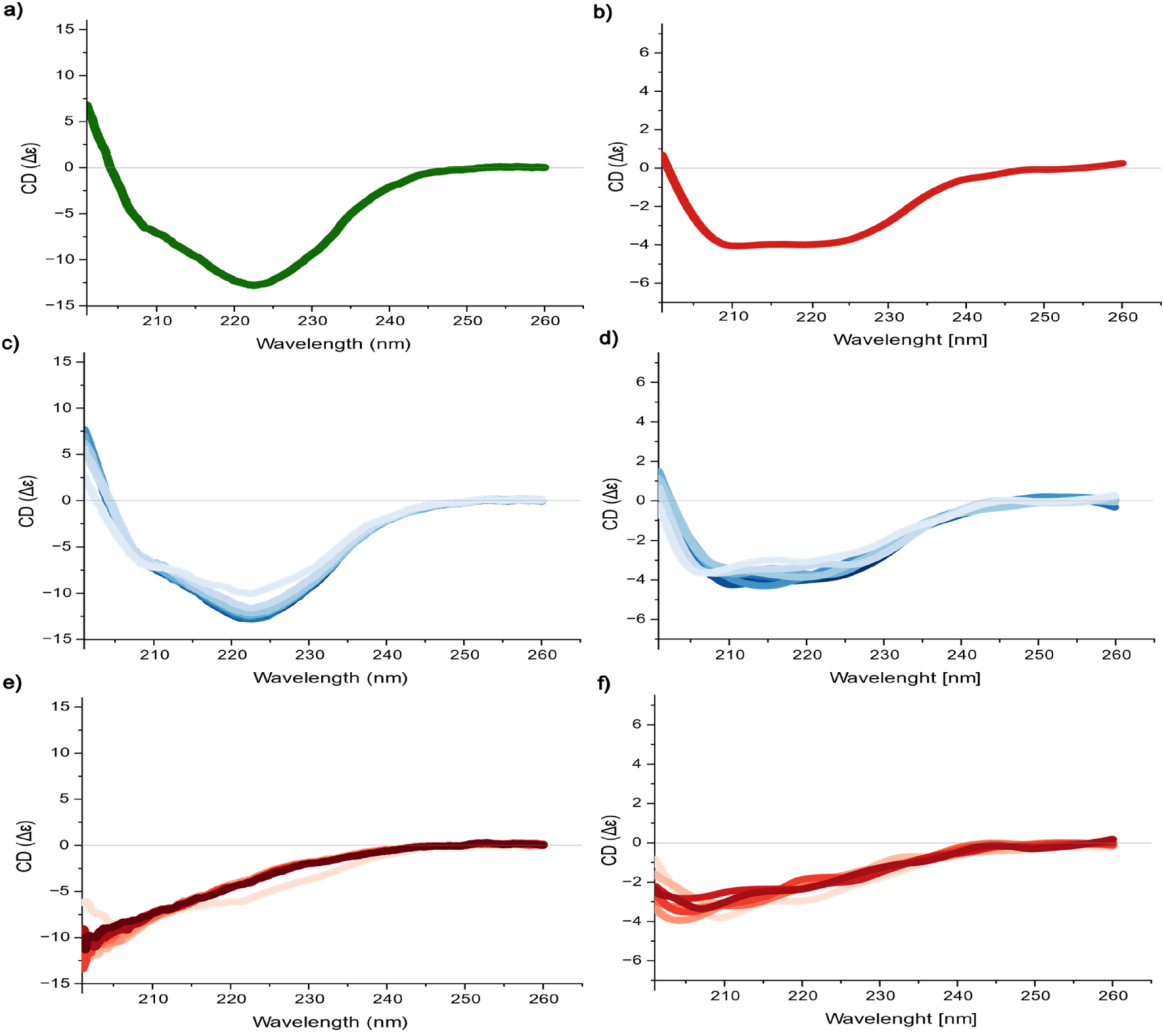
Circular dichroism (CD) spectra of (a) StmPr1 36 kDa (green) and (b) 47 kDa (red) and melting curves split 20–55 °C (shades of blue) and 60–90 °C (shades of red) for StmPr1 36 kDa (c, e) and 47 kDa (d, f).

StmPr1 is a member of the subtilases family defined by Siezen & Leunissen et al.^36^. It shows the main structural and functional elements of the family, such as a reactive triad consisting of Asp42, His105, and Ser289^22^. A single Calcium ion binds in a cleft at amino acids Asp5, Asp51, Gln116, Val121, and Met123. The oxyanion hole is located at Ser289 and Asn207 (Figs. 3 and 4) and two disulfide bridges support the structure stability (Figs. 1 and 4). Additionally, StmPr1 shows a broad substrate specifity^22^. To further analyze the place of StmPr1 in the subtilases family we used ESPript 3.0^37^ to create sequence alignments comparing StmPr1 to members of each sub-family (Fig. 4). The most homologous structure, regarding the sequence, is Thermitase^27^. The same holds for the structural alignment done via DALI server^38^ with a structural identity of 47%. While subtilisin Carlsberg^28^ and Proteinase K^26^ are close to that, both are structure-wise significantly less homologous with 42% and 38% respectively (Fig. 4a). Another indicator for the closer relationship to Thermitase^27^ is StmPr1’s thermal stability, as shown in Fig. 5c & e, StmPr1 is structurally stable up to 55 °C and arguably even up to 60 °C, considering the CD spectra we collected and analyzed from running a melting curve experiment using the J-815 CD spectrometer. Such increased heat resilience is a further indication of StmPr1 potentially belonging to the Thermitase family or being closely related to it. All these factors are valid not just for the 36 kDa variant but also for the 47 kDa variant. The values change, with Thermitase^27^ just having 35% and Furinsurpassing Proteinase K^26^ and subtilisin Carlsberg^28^ with 33%, over 29% and 32% of the strutures aligning respectively (Fig. 4b).

Meanwhile, resilience against heat is slightly higher with more of the secondary structure elements surviving to higher heat levels (Fig. 5d & f). The change in structural homology is a result of considering the addition of the 11 kDa C-terminus, missing in Proteinase K^26^ and subtilisin Carlsberg^28^. Such extensions are identified in other similar subtilases like Pro21717^29^ or BprV^39^ but are cut through the maturing process. That is not the case for StmPr1 which is excreted with a C-terminal extension leading to a 47 kDa version of StmPr1.

**Fig. 6:**
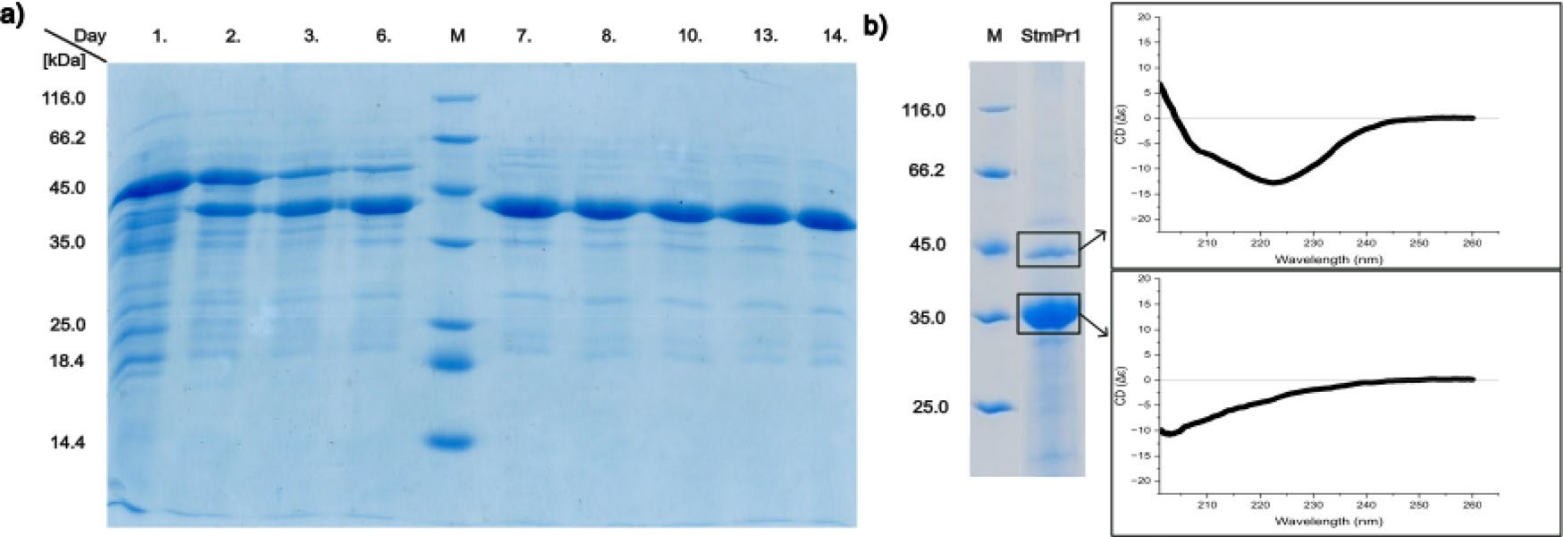
(a) SDS-PAGE of a 14-day self-digestion of StmPr1 from 47 kDa to 36 kDa at 20 °C. Showing the self-digestion process also observed for crystallization experiments. (b) SDS-PAGE of StmPr1 36 kDa showing both variants appearing in each expression of the 36 kDa StmPr1 plasmid and showing the corresponding CD spectra. SDS-PAGE in b) is cropped from the gel in the supplemental information.

The extension nevertheless is the target of autoproteolytic activity and over seven days at 20 °C 47 kDa StmPr1 digests itself to 36 kDa (Fig. 6).

The unique behavior and the C-terminal extension are indicators that StmPr1 may not be a part of the Thermitase family after all but is instead forming its own sub-family potentially in between Proprotein convertases and Thermitase.

### StmPr1 47 kda structural analysis

**Fig. 7:**
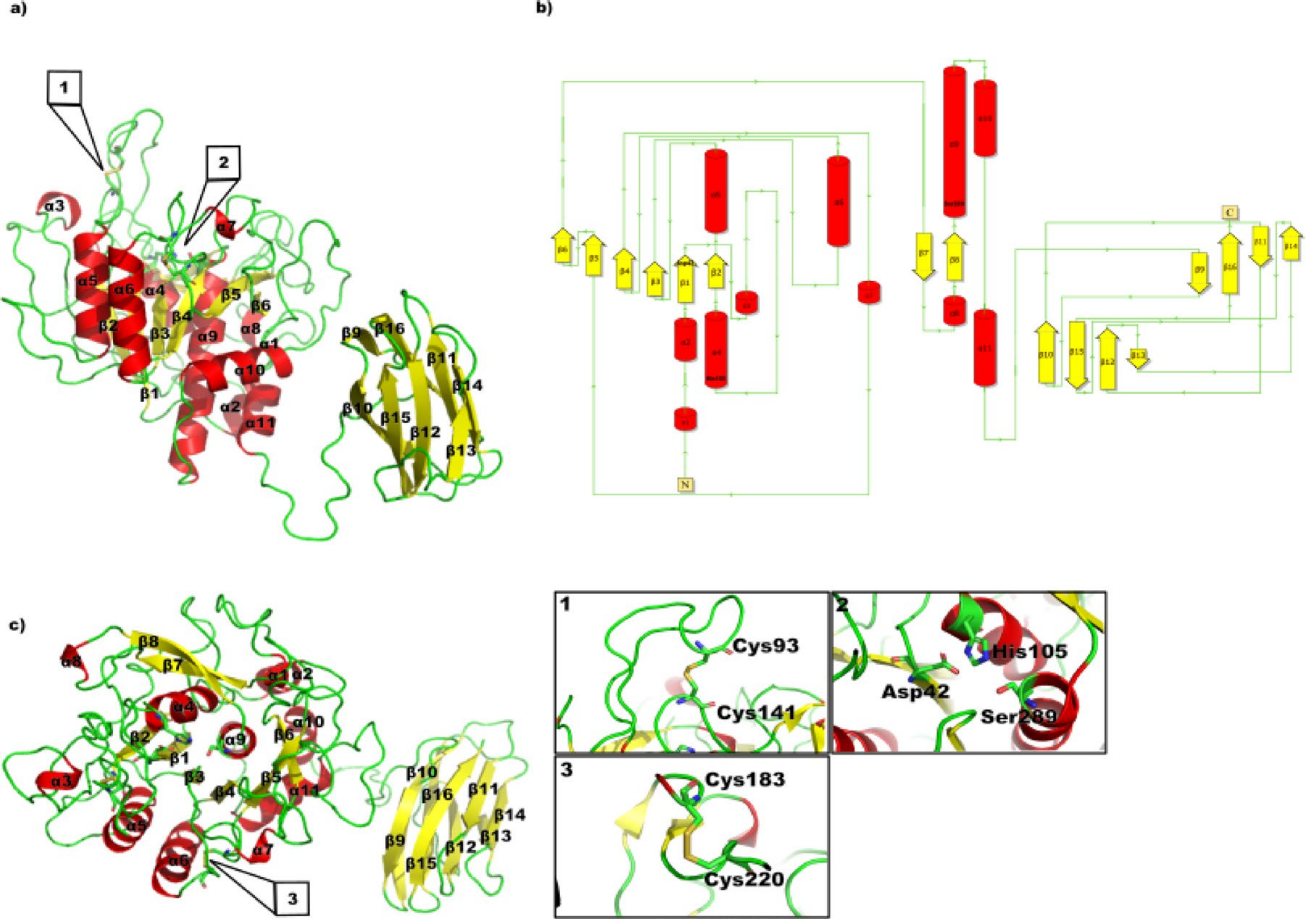
Cartoon and topology plot of StmPr1 47 kDa. (a) The secondary structure, predicted using AlphaFold3, is indicated in different colors. Loops are depicted in green, sheets in yellow and helices in red. (b) The topology plot is depicted in the same color scheme and highlights the Ca-binding site and catalytic triad. (c) Top view of the secondary structure indicated in the same colors to show secondary structure features missing in a). The two disulfide bridges (Cys93-Cys141, 1 and Cys183 - Cys220, 3) and the catalytic triad (2) are highlighted individual.

We used AlphaFold3 to predict the structure of the 47 kDa version of StmPr1, resulting in the structure presented in Figure 7. The structure shows some differences from the crystallographic obtained structure presented in Figure 1. The structure predicted by AlphaFold shortens most helices or sheets by one or two residues, but more importantly, several smaller structural formations, such as β2, β3, β6, and β8 of the 36 kDa structure, are missing. Equally missing are its α7 and α8 helices, while α1 is split into two helices by excluding Gln12. These variations are likely due to the inherent limitations of AlphaFold’s algorithm, which predicts residue torsion angles and may either overlook or inaccurately represent uncommon peripheral structures unique to StmPr1. This is primarily because its training data contains limited evolutionary examples of such structures and exhibits a bias toward ordered conformations. Consequently, the algorithm may incorrectly model helices within predominantly disordered regions. Therefore, the likelihood of AlphaFold predicting the smaller structures on the fringes of the model, which it misses examples for, is diminished^40–42^.

**Fig. 8:**
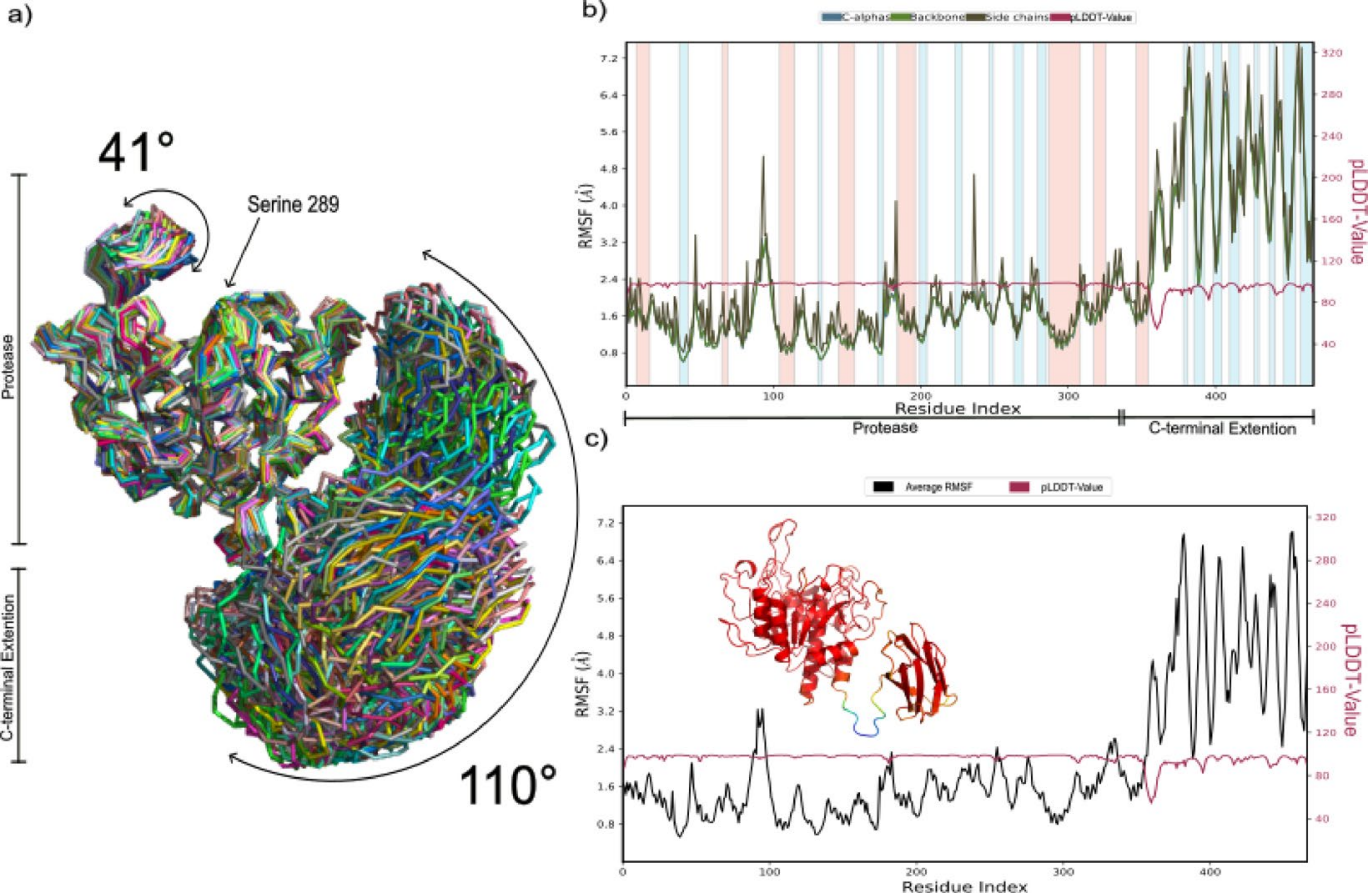
Flexibility analysis of StmPr1 36 kDa, Molecular Dynamics Simulation ran for 100 ns. (a) 250 frames extracted over the simulation superimposed. Delineations of flexible and rigid regions of the lid region over the catalytic triad and of the c-terminal Extension are annotated. (b) Root Mean Square Fluctuation (RMSF) and B-factor plots displaying changes in Cα, backbone, side chains, and heavy atoms for each residue. Different colors show different secondary structure elements: α-helices, blue, and β-sheets, pink. (c) Comparative B-factor plot derived from both, a predicted Local Distance Difference Test (pLDDT) Value data (received from the AlphaFold structure) and the MD simulation. The cartoon representation of StmPr1 47 kDa is color-coded to emphasize variations in pLDDT-Values, highlighting confidence in the accuracy of the predicted local structure. Simulations were done with Schoedinger’s Maestro Suite^33^.

Also, this was observed in slightly lesser pLDDT scores for the residues in the regions (Fig. 8b & c). Nevertheless, the main difference is the C-terminal extension consisting of eight anti-parallel coordinated β-sheets connected with small loop portions and linked to the main structure with a longer loop. The structure resembles the C-terminal extension of Furins with the sequence being slightly shorter.

**Fig. 9:**
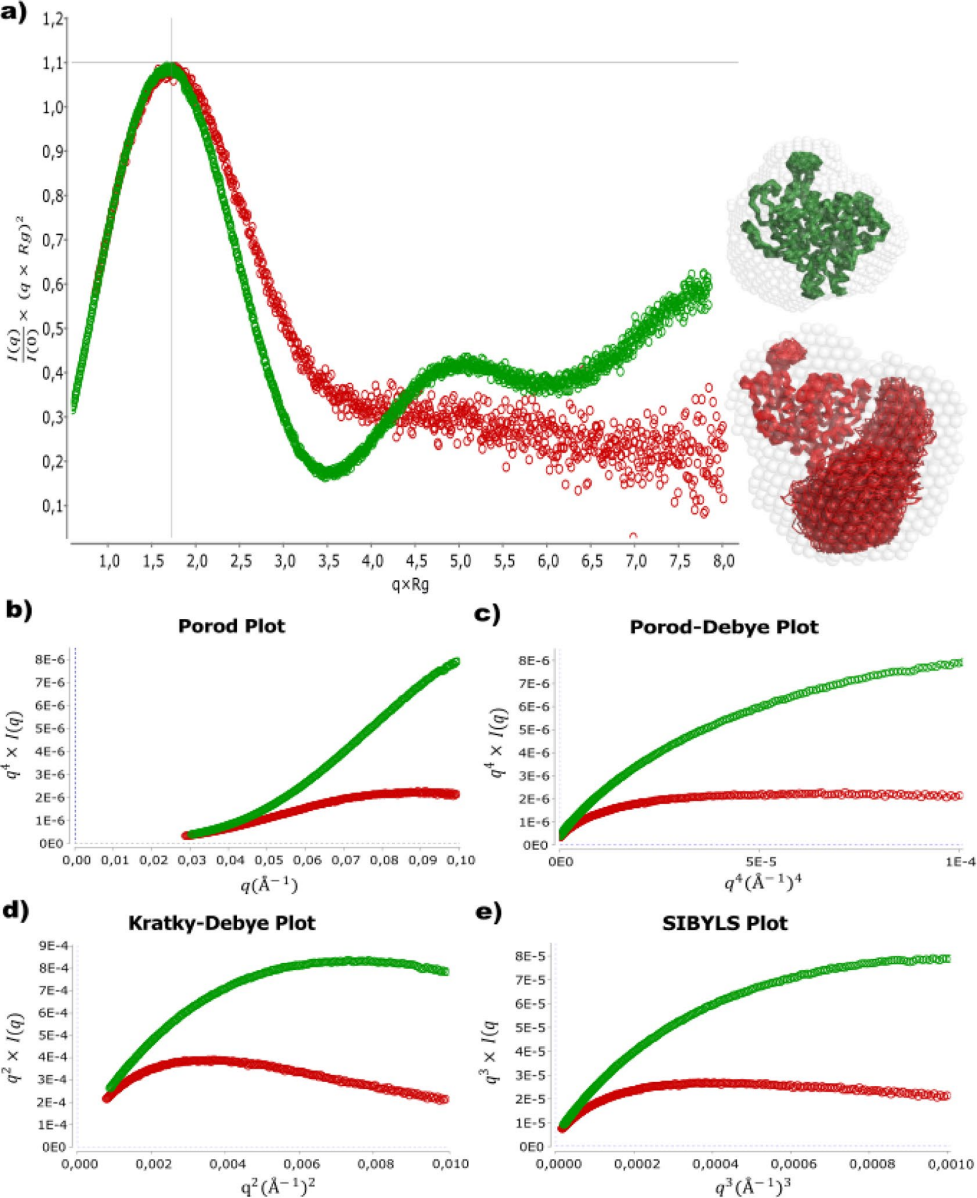
Exploiting the dimensionless Kratky plot and the Porod power-law relation to analyze the StmPr1 conformation in solution. The dimensionless Kratky plot with beat structures aligned to 250 aligned structures from a 100 ns Molecular Dynamics Simulation of StmPr1 36 kDa in green and 47 kDa in red, as well as SAXS data transformed (b) Porod plot (q4 I(q) vs. q), (c) Porod-Debye plot (q4 I(q) vs. q4), (d) Kratky-Debye plot (q2 I(q) vs. q2) and (e) SIBYLS plot (q3 I(q) vs. q3).

To further investigate the stability of the predicted structure we used MD simulations to simulate over 100 ns with the results presented in Fig. 8. While we found the structure to be reasonably stable (Fig. 8b & c) we also found that the C-terminal extension seems to be highly flexible, moving over a 110° angle (Fig. 8a). However, to confirm such in-silico data more evidence was needed. Hence, we decided to run small angle X-ray scattering (SAXS) experiments with both 36 kDa and 47 kDa protein to gain in-vitro evidence (Fig. 9). For both proteins, the Kratky plot showed a folded globular protein, while also revealing a higher flexibility of the 47 kDa variant with a slight shift to the right (Fig. 9a). The earlier point on which the curves for 47 kDa StmPr1 reach a plateau in the Porod, Porod-Debye, Krakty-Debye, and Sibyls plot further support the flexibility hypothesis of StmPr1s C-terminus region (Fig. 9b - e).

Finally, we used the ATSAS software package (version 3.1.3) to calculate the bead structures of both variants and aligned them with the structures received from the MD simulations to obtain the structures presented beside their curves in the Kratky plot (Fig. 9a). Not only did we receive globular structures, but structures also fitted the obtained simulations perfectly depicting the movement of the C-terminus over an approx. 110° angle. The structures predicted by AlphaFold thereby seem to be a good representation of the 47 kDa variant of StmPr1 and its C-terminal extension. Furthermore, this is the comparison of the CD-spectra, while both variants show a similar heat resilience, they also show a particular distinction. The curve for 36 kDa StmPr1 shows a negative peak at 222 nm, indicating a percentual higher count of α-helices in the structure^43^. This peak disappears in the 47 kDa curve, most likely covered by the stronger signal from the eight additional β-sheets present in 47 kDa StmPr1 (Fig. 5a & b).

The C-terminal extension corresponds to the bacterial pre-peptidase C-terminal domain (PPC, PF04151)^44^ which is also cataloged under the IPR007280 entry in the InterPro database^45^. Predominantly present in specific archaeal and bacterial proteases, this domain is typically located at the C-terminus of these enzymes. Its functions span various biological processes, including protein turnover, protein maturation, and stress response, as well as nutrient utilization, particularly in bacterial pathogens. In the latter context, the PPC domain contributes to virulence, underscoring its significance in pathogenic bacterial survival and adaptation.

StmPr1, as the primary virulence factor of *Stenotrophomonas maltophilia*, most probably has a significant role in nutrient acquisition. The PPC domain has been identified as a key contributor to collagen degradation, functioning as a binding site for collagen. This interaction is crucial for the bacterium’s pathogenicity, as it facilitates the breakdown of extracellular matrix components. This mechanism explains the observed induction of matrilysis and anoikis in human lung epithelial cells, as reported by DuMont & Cianciotto^46^. These processes are critical in tissue remodeling and bacterial invasion, further highlighting the virulence potential of StmPr1^47^.

When expressed using a plasmid only containing the sequence of 36 kDa StmPr1, missing the PPC region but still containing its N-terminal pro-peptide region, we could observe an interesting phenomenon. As shown in Fig. 6b, when run over an SDS-PAGE the active peak after SEC containing StmPr1 showed two bands. One wider band close to 35 kDa and one smaller closer to 45 kDa. We would expect a band closer to the 45 kDa band of the marker, regarding the self-digestion as shown in Fig. 6a. However, the majority of the protein seemed to run closer to the 35 kDa band. We ran the sample through IEX to separate the bands and the result was an active fraction corresponding to the upper band and a non-active fraction corresponding to the lower band. Performing for both CD measurements we obtained the spectra presented in Fig. 6b, showing that the lower band protein was not correctly folded, resulting in a non-active fraction of the protein.

These results underscore another function of the PPC region, which also acts as an intramolecular chaperon guiding the correct folding of the enzyme^48^. Considering those functions of the C-terminal extension proposed for StmPr1, a natural occurrence of the self-digestion observed under lab conditions is most probably unlikely. More likely is, that it is an artifact observed under lab conditions, resulting from relatively high protein concentrations, rather than a natural maturation process. Nevertheless, the occurrence of a post-excretion PPC region till now is rarely observed for alkaline serine proteases like StmPr1 or KP-43, another alkaline serine protease identified to have a post-excretion PPC region^49^.

## Conclusion

StmPr1 displays several unique structural and functional elements, distinguishing it from other members of the Subtilase family. Its flexibility and thermostability are significantly affected by specific β-sheet formations and α-helices located at the surface of the protein. Especially the inclusion of β6/β8 and α7, resemble those observed in the cold-active protease Pro21717, suggesting an extended temperature tolerance. This is further supported by its calcium-binding side and disulfide bridges, providing additional stability. Molecular dynamic simulations indicate a substrate trapping and inhibitor protection activity of the described lid region, formed by the loop connecting β1 and α5. StmPr1’s C-terminal extension appears to play a significant role in the protein’s functional resilience and proper folding. The flexibility of the PPC region, indicated by molecular dynamics and further supported by SAXS experiments, indicates a crucial role in its proteolytic activity by facilitating distinct substrate interactions. The PPCs’ relevance for the correct folding of StmPr1 is shown by the presence of misfolded and non-functional protein variants when the C-terminus is absent. These observations and data highlight StmPr1’s evolutionary adaptations and functional advantages within the subtilase family and the relevance of StmPr1 as a potential therapeutic target for the treatment of *S. maltophilia* infections. In this context our inhibitor studies presented provide interesting candidates for further drug discovery and drug design investigations. Particularly with Bortezomib, we provide an interesting candidate for further investigation regarding a *S. maltophilia* therapy.

## Materials and methods

### Molecular cloning, protein expression, and purification

The *stmpr1* gene was cloned into a pET17b plasmid by BioCat GmbH, additionally, a sequence of the gene missing the sequence for the C-terminal extension of StmPr1 was cloned in a pET28a plasmid. Both plasmids were induced in chemically competent E. coli BL21 DE3 cells. These cells were grown in LB media containing 50 µg/ml ampicillin at 37 °C until reaching an optical density (OD_600_) of 1.0. After reaching the right OD_600_, 1 mM IPTG was added to the cells, which were grown at 20 °C for an additional 48 h. Following the growth period, the cells were separated from the supernatant by centrifugation at 12,000 x g. The supernatant with the excreted 36 kDa and 47 kDa StmPr1 protein was separated from the cell pellet and concentrated via tangential flow filtration. The resulting concentrate was precipitated using 75% w/V ammonium sulfate. The precipitation was centrifuged at 17,000 x g and the resulting pellet resuspended in a buffer comprising 20 mM Tris-HCl, 100 mM NaCl, and adjusted to pH 8.0. The protein solution was dialyzed overnight using the same buffer to get rid of the remaining ammonium sulfate. The following step was size exclusion chromatography. Resulting fractions were assessed for activity using N-Succinyl-Ala-Ala-Pro-Phe-p-nitroanilide as substrate. Active fractions were pooled, dialyzed overnight with a buffer of 20 mM Tris-HCl at pH 8, and further purified using ion exchange chromatography. Peak fractions matching the protein, evaluated via substrate again, were pooled and concentrated to a final concentration of 10 mg/ml using a 30 kDa Amicon concentrator.

### Protein crystallization

Crystallization experiments were done with a monodisperse solution of StmPr1 47 kDa, dispersity was analyzed before crystallization experiments using dynamic light scattering. Crystals suitable for X-ray diffraction were obtained by hanging drop vapor diffusion at 20 °C. Therefore, 1 µl of protein solution was mixed with 1 µl of 1.8 M ammonium sulfate, and 0.1 M Tris at pH 8. Crystals used for experiments with inhibitors were prepared using 0.9 M ammonium sulfate, 0.4 M lithium sulfate, and 0.1 M sodium citrate at pH 6. In ligand binding experiments, both co-crystallization and soaking experiments were performed. Different buffers were required for soaking experiments, Bortezomib, Chymostatin, and Leupeptin were solved and soaked using 0.1 ml of 100 mM solution with dimethylsulfide (DMSO). The combinations were set against a 1 ml reservoir solution of identical composition. Crystals were observed after approx. 60 days. Through those 60 days StmPr1 47 kDa digests itself to 36 kDa by cleaving its C-Terminus. Before data collection crystals were cryo-protected with a solution having the conditions where the crystals were grown in, containing an additional 12% glycerol. Crystals were soaked for 1 h in a solution containing 100 mM inhibitor solved in water for PMSF and solved in DMSO for Bortezomib, respectively.

### Diffraction data collection and structure determination

Native X-ray diffraction data were collected at 100 K at beamline P13 (PETRA III, DESY)^50^. Data were afterward processed with the XDS program package (version 2010)^51^. All Data collection and refinement parameters are summarized in Table 1. To solve the phase problem Subtilisin Carlsberg (PDB ID: 1YU6) was used as a search structure using the Phaser-MR’s one-component interface program embedded within the Phenix software suite (version 1.19.2–4158)^52,53^. Structure refinement was done with the Phenix.refine tool and REFMAC (version 5.8.0091)^53–56^. Structure modeling was done with WinCoot 0.9.8.7.1^57^.

### SAXS data collection and analyses

SAXS data were collected at the beamline P12 (PETRA III, DESY) at a temperature of 293.15 K employing the in-batch measurement approach. Starting with a protein concentration of 2 mg/ml for StmPr1 47 kDa and 5 mg/ml for 36 kDa, and systematically serial dilutions were done to achieve concentrations as low as 0.2 mg/ml for 47 kDa and 0.5 mg/ml, using the dialysis buffer as the diluent. The results were subsequently processed and visualized using the BioXTAS RAW software (version 2.3.0), complemented by further analysis with ATSAS GNOM (version 3.1.3)^58,59^.

### Activity assay measurements and analyses

The protease activity of StmPr1 was determined using an adapted protocol established by Windhorst et al.^22^. Not changing buffer composition, protein concentration was set at 10 nM, and the concentration of the substrate was set at 1.4 mM. Both were added to the preprepared well using the Te-InjectTM-reagent injector of Tecan’s Infinite 200 PRO multimode reader, the protein was incubated for 10 min with the inhibitor before adding the substrate.

### Molecular dynamics simulation

For our molecular dynamic simulation studies, the crystallographic structure of StmPr1 36 kDa and the structure predicted by AlphaFold 3 for StmPr1 47 kDa were used. Both structures were meticulously prepared using the protein preparation workflow integrated within Schroedinger’s Maestro suite (version 01–2023)^33^. Initially, hydrogen atoms were added to ensure the chemical integrity of the structures. The hydrogen-bonding network was refined using PROPKA, using predicted pKa values and the protein’s local environment to optimize H-bond assignments. A subsequently restrained minimization was conducted using the OPLS4 force field, with crystallographic waters beyond the first solvation shell being removed^60^. The solvated system for the molecular dynamics (MD) simulation was built using the TIP4P water model and enclosed within an orthorhombic periodic boundary box^61^. A minimum of 20 Å from the protein to the edges of the box was maintained to prevent direct interactions between periodic images. Further, a salt concentration of 150 mM NaCl was used to mimic physiological conditions. Ensuring a constant particle number, a constant pressure of 1.01325 bar, and a constant temperature of 310.15 K the NPT ensemble was kept for the whole integration conducted over a trajectory of 100 ns saving configurations every 20 ps resulting in 250 frames to use in the analysis for each structure^62^.

### CD measurements

StmPr1 solution concentrations for the experiments were between 0.05 and 0.2 mg/ml and measured over a wavelength range of 260 nm to 190 nm. The samples were measured 15 times, the buffer was measured 5 times and respective spectra were merged. The final spectra presented are the results of the buffer spectra being subtracted from the protein spectra. Spectra were measured at 20 °C. For the melting curves, the temperature range was set from 20 °C to 90 °C, rising in 5 °C steps. Each temperature step was measured 3 times and merged. Measurements were done by applying a J-815 CD Spectropolarimeter (Jasco, Tokyo, Japan).

### Graphics

Molecular graphics were generated using PyMOL (The PyMOL Molecular Graphics System, Version 2.5, Schrödinger, LLC).

## Electronic supplementary material

Below is the link to the electronic supplementary material.

Supplementary Material 1

## Data Availability

All structural data presented in this study is deposited in the Protein Data Bank archive (https://www.rcsb.org/) and is available via accession codes: 9G8V, 9GOI, 9GRG, 9I6C and 9I67. SAXS data presented in this study is deposited in the Small Angle Scattering Biological Data Bank (https://www.sasbdb.org/) and is available via accession codes: SASDXK3 and SASDXK3. Further details are available in the Supplementary. All other data obtained in this study are available from the corresponding authors upon request.
