# Supplementary material for: Unveiling the structure, function and dynamics of StmPr1 in Stenotrophomonas maltophilia virulence
Source: Sci Rep. 2025 Jun 20;15:20193. doi: 10.1038/s41598-025-06177-5 (PMC12181431; doi:10.1038/s41598-025-06177-5)
Supplement: Supplementary file 1 — Supplementary Material 1 [file 41598_2025_6177_MOESM1_ESM.docx]

**Supplemental information**
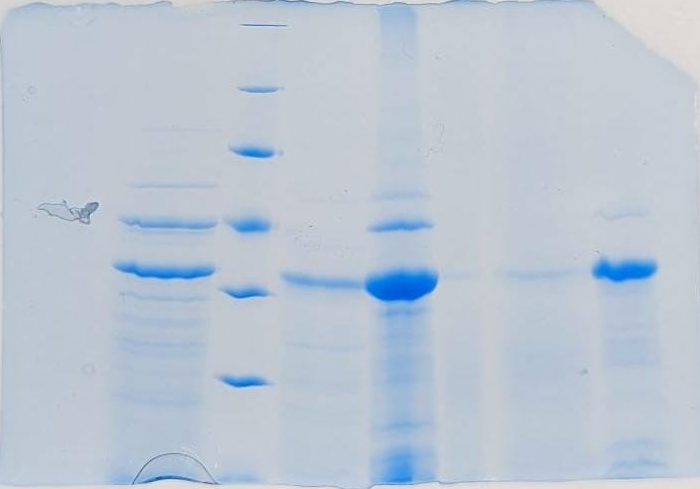


Supplemental 1: Original gel of the cropped gel presented in Figure 6.


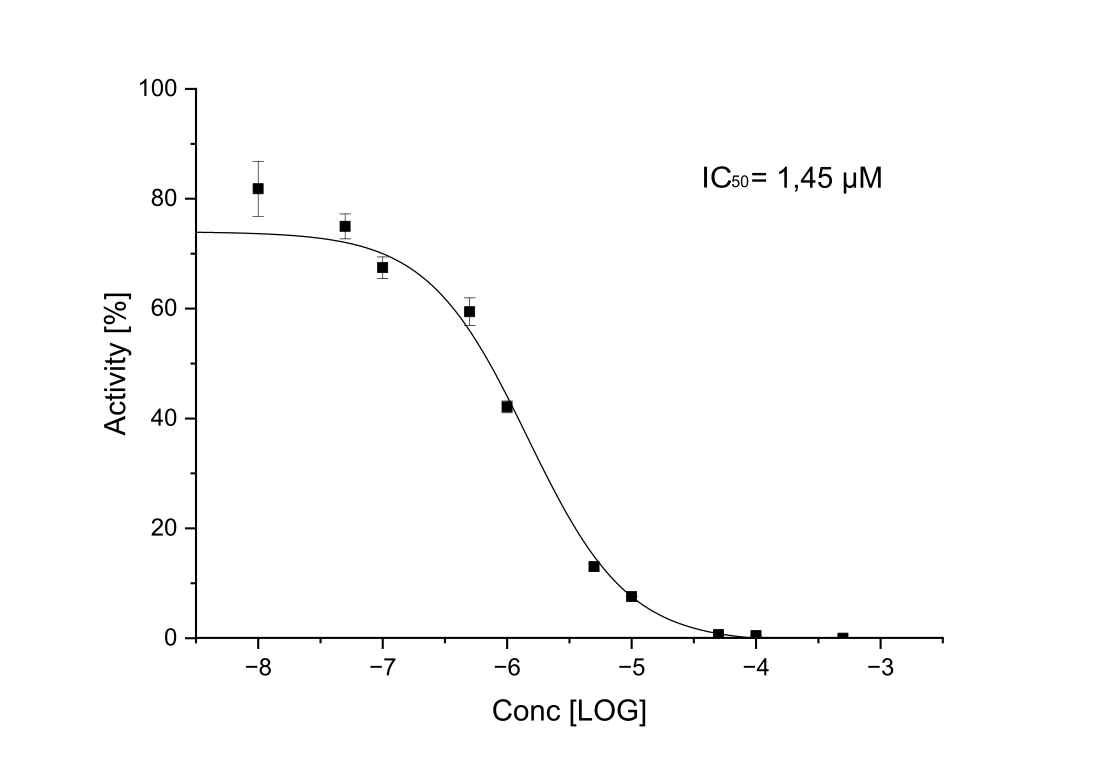


Supplemental 2: Activity curve shows Proteinase K activity and IC_50_ against Bortezomib.
